# Health outcomes of non-nutritive sweeteners: analysis of the research landscape

**DOI:** 10.1186/s12937-017-0278-x

**Published:** 2017-09-08

**Authors:** Szimonetta Lohner, Ingrid Toews, Joerg J. Meerpohl

**Affiliations:** 1 Cochrane Hungary, Medical Center, University of Pécs, Pécs, Hungary; 2 Cochrane Germany, Medical Center - University of Freiburg, Faculty of Medicine, University of Freiburg, Breisacher Str. 153, Freiburg, 79110 Germany; 3 Centre de Recherche Épidémiologie et Statistique Sorbonne Paris Cité – U1153, Inserm / Université Paris Descartes, Cochrane France, Hôpital Hôtel-Dieu, 1 place du Parvis Notre Dame, 75181 Paris, Cedex 04 France

**Keywords:** Non-nutritive sweetener, Artificial sweetener, Aspartame, Saccharin, Stevia, Diabetes, Cancer, Dental caries, Weight gain, Overweight, Obesity, Scoping review

## Abstract

**Background:**

Food products containing non-nutritive sweeteners (NNSs) instead of sugar have become increasingly popular in the last decades. Their appeal is obviously related to their calorie-free sweet taste. However, with the dramatic increase in their consumption, it is reasonable and timely to evaluate their potential health benefits and, more importantly, potential adverse effects. The main aim of this scoping review was to map the evidence about health outcomes possibly associated with regular NNS consumption by examining the extent, range, and nature of research activity in this area.

**Methods:**

We systematically searched Ovid MEDLINE, EMBASE and the Cochrane CENTRAL databases for studies on NNSs (artificial sweeteners or natural, non-caloric sweeteners, either used individually or in combination) using text terms with appropriate truncation and relevant indexing terms. All human studies investigating any health outcomes of a NNS intervention or exposure were eligible for inclusion. No studies were excluded based on language, study design or methodological quality. Data for each health outcome were summarized in tabular form and were discussed narratively.

**Results:**

Finally, we included 372 studies in our scoping review, comprising 15 systematic reviews, 155 randomized controlled trials (RCTs), 23 non-randomized controlled trials, 57 cohort studies, 52 case-control studies, 28 cross sectional studies and 42 case series/case reports.

In healthy subjects, appetite and short term food intake, risk of cancer, risk of diabetes, risk of dental caries, weight gain and risk of obesity are the most investigated health outcomes. Overall there is no conclusive evidence for beneficial and harmful effects on those outcomes. Numerous health outcomes including headaches, depression, behavioral and cognitive effects, neurological effects, risk of preterm delivery, cardiovascular effects or risk of chronic kidney disease were investigated in fewer studies and further research is needed. In subjects with diabetes and hypertension, the evidence regarding health outcomes of NNS use is also inconsistent.

**Conclusions:**

This scoping review identifies the needs for future research to address the numerous evidence gaps related to health effects of NNSs use.It also specifies the research questions and areas where a systematic review with meta-analyses is required for the proper evaluation of health outcomes associated to regular NNSs consumption.

## Introduction

In the last decades, growing concerns about health and quality of life have encouraged people to avoid the consumption of food rich in sugar, salt or fat [1, 2]. With increased consumer interest in reducing sugar intake, food products containing calorie-free alternatives (non-nutritive sweeteners; NNSs) have become increasingly popular [3, 4]. NNSs are generallyseveral hundred to several thousand times sweeter than sucrose [5]. Most of them do not contain any calories while some NNSs (e.g. aspartame) contain very few [6]. Each sweetener has specific characteristics of sweetness intensity, persistence of the sweet taste, coating of the teeth and aftertaste effect [7, 8].

Most of the NNSs approved for human consumption are synthetic (artificial sweeteners; AS). However, more and more NNSs of natural origin are available on the market (natural, non-caloric sweeteners; NNCSs). The most familiar NNCSs are *Stevia rebaudiana*-based products. Steviol glycosides, extracted from the plant Stevia include stevioside and rebaudioside A, but also other, less common glycosides [9].

With regard to the range of approved ASs there are differences among countries. In the United States for example, there are currently six ASs which the Food and Drug Administration (FDA) has approved for consumption (Table 1; [10] acesulfame-K, aspartame, neotame, saccharin, sucralose and advantame). In the European Union meanwhile, the range of currently approved ASs is wider, also including, for example, cyclamate [11, 12]. Stevia has been used as a sweetener for decades in some countries (e.g. Japan), while it was approved as a food additive just recently by the European Food Safety Authority (EFSA) [13] and the US FDA.

**Table 1:** Non-nutritive sweeteners available in the USA and the European Union, and their Acceptable Daily Intake levels, as defined by regulatory bodies

	Acceptable Daily Intake defined by the FDA (mg/kg bw)	Acceptable Daily Intake defined by the SCF/EFSA (mg/kg bw)
ACE K	15	9
Advantame	32.8	5
Aspartame	50	40
Cyclamate	not approved	7
Luo Han Guo fruit extracts	not specified	not specified
Neohesperidine DC	not approved	5
Neotame	0.3	2
Saccharin	15	5
Sucralose	5	15
Steviol glycosides	4	4
Thaumatin	not approved	not specified

*Abbreviations*: *EFSA* European Food Safety Authority, *FDA* Food and Drug Administration, *SCF* Scientific Committee on Food (European Commission)

Parallel to the dramatic increase in the consumption of food and beverages sweetened with NNSs, concerns have been raised about their potential adverse health effects [14–16]. Several studies investigated short-term consequences (e.g. on food intake, mood, blood pressure); others evaluated long-term health effects (e.g. on body weight, incidence of obesity, risk of cancer, risk of diabetes or dental caries) of NNSs. Overall, plenty of scientific studies have been published, postulating a wide variety of beneficial, but also negative health effects of NNSs.

Since scoping reviews are used to present a broad overview of the evidence pertaining to a topic irrespective of study quality, they can be seen as a hypothesis-generating exercise and are therefore the optimal method for examining this emerging area as a first approach [17]. The aim of this scoping review was to map the available evidence about the health outcomes possibly associated with regular NNS consumption by examining the extent, range, and nature of research activity in this area.

### Objectives

Primary objectives of this scoping review were to:

- Identify all potential health outcomes associated with regular NNS consumption;
- Define the number and types of primary studies (i.e. studies that collect original data from subjects) available for each health outcome;
- Identify any gaps in the evidence base for the health outcomes of regular NNS consumption.

Secondary objective of this scoping review was to:

- Summarize available systematic reviews on the association of NNS consumption and health outcomes, compare their inclusion criteria and limitations, and determine whether a new systematic review in this area is justified.

## Methods

We used the approach of a scoping review (including a process known as evidence mapping) [18, 19] to compile all relevant evidence about the health effects of NNS consumption from the scientific literature. This approach is based on a systematic literature search and the transparent assessment of the retrieved evidence for its relevance for the research question by presenting an overview of a potentially large and diverse body of literature pertaining to this broad research topic, without making restrictions based on study design and methodology. Furthermore, it seeks to provide a descriptive summary of the evidence without detailed critical appraisal of included individual studies.

### Inclusion criteria

To be included, a primary study needed to meet all of the following criteria: a) a study on human beings (of any age, gender or health status); b) an intervention with or exposure to any type and any dosage of ASs (aspartame, acesulfame potassium, saccharin, sucralose, advantame, neotame, cyclamate, alitame, neohesperidin dihydrochalcone (DC)) or NNCSs (stevioside, rebaudioside A, thaumatin, brazzein) or NNSs (defined as any combination of AS and NNCS); c) a study reporting health effects of any type (both health outcomes and intermediate markers of health outcomes were included); d) no restriction on study design or language.

We also included relevant systematic reviews on the association of an NNS intervention/exposure and one or more defined health outcomes (every review describing or indicating a systematic search was regarded to be a systematic review).

In this manuscript we report on relevant systematic reviews, clinical trials, cohort studies, case-control and cross-sectional studies.

### Search strategy

Ovid MEDLINE (ovidsp.ovid.com), EMBASE (www.embase.com) and the Cochrane CENTRAL database (www.cochranelibrary.com) were searched from inception to October Week 2 2015 for studies on AS and to January Week 3 2016 for studies on NNCS and NNS, using text words with appropriate truncation and relevant indexing terms (MeSH). The search was in the form [terms for artificial sweeteners/ natural, non-caloric sweeteners/non-nutritive sweeteners] and [human studies]. Electronic searches were limited neither in time nor in language. Electronic searches were followed by hand searching of reference lists of relevant review articles and included primary studies. Electronic searches were updated in May Week 4 2017.

### Data extraction and management

Titles and abstracts were screened for inclusion by a single reviewer (SL). Only clearly irrelevant records were excluded at this stage. All potentially relevant abstracts and full papers were screened for inclusion by two reviewers independently using an inclusion/exclusion form specifically developed for the purpose of this scoping review (SL and IT). In case of disagreement, the subject was discussed among the two reviewers until a mutual decision could be made. When this was not possible, a third reviewer (JM) was consulted. A data extraction sheet was designed and piloted. Then two reviewers (SL and IT) independently extracted the following data for each included primary study: 1) first author; 2) year of publication; 3) study location; 4) study design; 5) aim of the study; 6) main characteristics and size of the study sample; 7) main characteristics of intervention/exposure and control; 8) outcome measures with direction of effect.

Intervention studies were classified as RCTs (with either parallel, or cross-over design) or non-randomized controlled trials (non-RCTs), while observational studies were classified as prospective or retrospective cohort studies, cross-sectional studies, case-control studies, ecological studies or case reports/case series. Data sheets were compared and in case of differences in the extracted data, the relevant information was checked again in the study article and corrected.

Data for each health outcome were summarized in tabular form and were discussed narratively. Bubble charts were used to highlight the main relationship among the types of NNS used in the studies as intervention/exposure, the health effects and the study types. Bubble charts are multi-variable graphs, whose plot points along a grid where the X and Y axis are separate variables (in our case they represent the type of sweetener and health outcomes).Additionally, the different colours of the plotted points represent a third variable (in our case they show the study type).

For each included systematic review following data were extracted: 1) first author; 2) year of publication; 3) date of search; 4) databases searched 5) aim of the review; 6) study design of eligible studies; 7) main characteristics of eligible intervention/exposure; 8) outcome(s) eligible for inclusion.

## Results

**Fig. 1:**
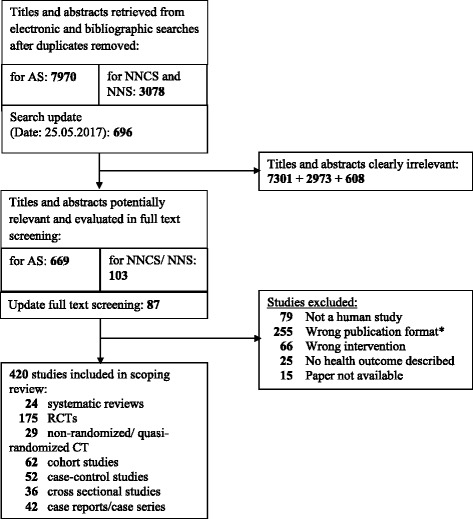
Flow diagram for the systematic search on artificial sweeteners, natural non-caloric sweeteners and non-nutritive sweeteners. *All manuscripts which described neither a primary study nor were systematic reviews (e.g. narrative summaries, commentaries, and letters) were excluded as “Wrong publication format”

The flow diagram of the literature search (PRISMA Flow Diagram adapted for the scoping review process) is shown in Fig. 1. For ASs a total of 7970 articles were identified in the initial literature search, of which 669 appeared to be potentially relevant. Fifteen papers could not be retrieved; all others were available for detailed full-text assessment. Finally, 317 articles fulfilled the inclusion criteria. This search focused on studies with ASs as the intervention or exposure; however, 11 primary studies with NNSs, 28 studies with diet beverages/diet sodas and one study with a combination of NNSs and sugar-alcohols were already identified at this stage. For NNCSs and NNSs, 3087 articles were identified in the original literature search, 112 full texts were screened for eligibility and finally 55 were included in the review. In 2017, after the update search of databases, 48 further studies were eligible for inclusion.

In total, 24 systematic reviews (Table 2), 175 randomized controlled trials (RCTs), 29 non-randomized controlled clinical trials (non-RCTs), 62 cohort studies, 52 case-control studies and 36 cross-sectional studies were included in this scoping review. We also found 42 case studies.

**Table 2:** Systematic reviews investigating health effects of non-nutritive sweeteners

First author, publication year	Population	Intervention/ Exposure	Outcome	Included study designs	Limitations	Date of search	Searched databases
Bernardo, 2016 [274]	adults and children	AS use	adverse clinical effects	comparative and epidemiological studies	ND	ND	MEDLINE; EMBASE; Cochrane Library; Lilacs/Scielo
Berry, 2016 [84]	ND	sucralose consumption	carcinogenic potential	ND	ND	ND	MEDLINE; TOXFILE, BIOSIS Toxline; FOODLINE; CAB Abstracts; Food Science and Technology Abstracts; NTIS; EMBASE
Borkum, 2016 [275]	ND	migraine triggers (including aspartame)	oxidative stress in the brain	ND	published between1990–2014 and in English language	ND	MEDLINE
Brown, 2010 [22]	children (0–18 y)	AS consumption	metabolic health effects (food intake, weight change, diabetes, metabolic syndrome components)	ND	published in peer reviewed journals in English language; published full text available	ND	MEDLINE, Web of Science, EMBASE
Greenwood, 2014 [157]	generally healthy population	sugar- or artificially-sweetened beverage consumption	incident diabetes mellitus type 2 risk	prospective observational studies (min. Duration: 3 years)	published since 1990 and in English language	November 2009; updated: June 2013	Cochrane Library; MEDLINE; MEDLINE In-Process; EMBASE; CAB Abstracts; ISI Web of Science; BIOSIS
Cheungpasitporn, 2014 [135]	ND	sugar- or artificially-sweetened soda consumption	chronic kidney disease incidence	RCTs, case–control, cross-sectional or cohort studies	provided odds ratios, relative risks, hazard ratios or standardized incidence ratios with 95% confidence intervals	June 2014	MEDLINE, EMBASE, Cochrane Library, CENTRAL
Hendriksen, 2011 [276]	ND	added sugar and intense sweeteners	beneficial and hazardous health effects	ND	written in English or Dutch language	October 2008	ND
Imamura, 2016 [161]	adults without diabetes	artificially sweetened beverages	incidence of type 2 diabetes	prospective studies	no language or time limitations	May 2013; updated: February 2014	MEDLINE; EMBASE; Ovid; Web of Science
Miller, 2014 [181]	generally healthy population	low-calorie sweeteners from foods or beverages or as tabletop sweeteners	body weight or body composition	RCTs and prospective cohort studies	a minimum study duration of 2 weeks for RCTs and 6 months for prospective cohorts	September 2013	MEDLINE
Pereira, 2014 [180]	no limitation	ASB (or sugar- sweetened beverages) consumption	body weight or body fat	RCTs and prospective cohort studies	observational studies min. Duration of 6 months	March 2012	MEDLINE
Pereira, 2013 [277]	ND	DB/ASB consumption	body weight, obesity risk, type 2 diabetes, or cardiovascular disease	ND	studies in English language	September 2011	MEDLINE
Reid, 2016 [183]	pregnant women, infants, or children (<12 years of age)	early life NNS exposure (all types of NNS consumption)	long-term metabolic health (BMI, birth weight, growth velocity, incidence of overweight/ obesity, change in adiposity, incidence of impaired glucose tolerance, metabolic syndrome, insulin resistance or type 2 diabetes)	RCTs and prospective cohort studies	min. Study duration of 6 months	July 2015	MEDLINE; EMBASE; Cochrane Library
Rogers, 2016 [182]	humans and animals	low-energy sweeteners consumption	energy intake, body weight, BMI	ND	no language or time limitations	February 2015	MEDLINE, EMBASE, Web of Science
Romo-Romo, 2016 [24]	adults	NNS consumption	glucose metabolism and appetite regulating hormones, development of metabolic chronic diseases	observational studies and clinical trials	follow up of at least 3 years in cohort studies	April 2015; updated: March 2016	MEDLINE, Cochrane Library, Trip Database
Russel, 2016 [278]	adult type 2 diabetes patients or obese subjects	nutrients (incl. Low-calorie sweeteners)	postprandial hyperglycemia	intervention trials	studies in English language	ND	MEDLINE, Web of Science
Shankar, 2013 [279]	ND	NNS consumption	obesity/weight gain; diabetes; cardiometabolic indicators	ND	ND	2012	MEDLINE
Spencer, 2016 [280]	humans and animals	aspartame, saccharin or sucralose consumption	fermentation, absorption, gastrointestinal symptoms	ND	full articles in English language	June 2015	MEDLINE, EMBASE
Timpe Behnen, 2013 [281]	diabetes patients	acesulfame, aspartame,luo han guo, monk fruit, neotame, rebiana, saccharin, stevia, and sucralose	diabetic control, including, but not limited to, blood glucose levels, postprandial blood glucose, HbA1c	clinical studies	studies in English language	May 2012	MEDLINE, Scopus
Wiebe, 2011 [23]	ND	a sweetener (e.g. non-caloric sweetener)	weight change, energy intake, lipids, HbA1C, insulin resistance	parallel or crossover RCT	follow-up at least 1 week in duration; at least 10 participants per group, no trials with placebo control	January 2011	MEDLINE, EMBASE, Cochrane Library CENTRAL, CAB Global
Oliver, 2015 [85]	ND	aspartame, ace-K, cyclamic acid and its salts, steviol glycosides, neohesperidin DC, neotame, saccharine and its salts, sucralose,aspartame-acesulfame salt, thaumatin	benefits and risks related to intense sweeteners	meta-analysis, RCTs, quasi experimental, cohort, case-control, cross-sectional studies	none	ND	MEDLINE, Cochrane Database of Systematic Reviews, Psychinfo
Onakpoya, 2015 [21]	adult volunteers (>18 y)	steviol glycoside	cardiovascular risk factors (blood pressure, blood sugar, cholesterol)	double-blind RCTs	No age, language or time restrictions. Studies in which steviol glycosides were combined with other dietary supplements were excluded	May 2014	MEDLINE, EMBASE, Amed, Cinahl, The Cochrane Library, Google Scholar
Poolsup, 2012 [282]	patients with hypertension	stevioside	systolic and diastolic blood pressure control	RCTs	published in English language	February 2012	MEDLINE, Science Direct, Cochrane Library, Wiley Online Library
Ulbricht, 2010 [20]	both adults and children	stevia	adverse effects, (pharmacology, kynetics, dosing, interactions, toxicology)	no restriction (both in vivo and in vitro studies)	no language restrictions	ND	AMED, CANCERLIT, CINAHL, CISCOM, Cochrane Library, EMBASE, HerbMed, International Pharmaceutical Abstracts, MEDLINE, NAPRALELT
Urban, 2015 [283]	ND	steviol glycosides and/or stevia leaf extracts of known concentrations	allergic reactions	no restriction (also animal and in vitro studies)	ND	October 2014	MEDLINE, Science Direct, Google Scholar
Wang, 2016 [284]	adults, pregnant women and infants (>6 mo)	FDA-approved sweeteners	energy sensing by the brain; gut hormones that may influence energy homeostasis; satiety and preference f r taste; eating behavior; body weight and composition	RCTs, non-RCT, not controlled trials, prospective cohorts	English language; cancer patients were excluded	ND	MEDLINE

*Abbreviations*: *ASB* artificially sweetened beverage, *DB* diet beverage, *HbA1c* glycosylated haemoglobin type A1C, *ND* not described, *RCT* randomized controlled trial; y, years; mo, months

### Health outcomes assessed in the included studies

**Fig. 2:**
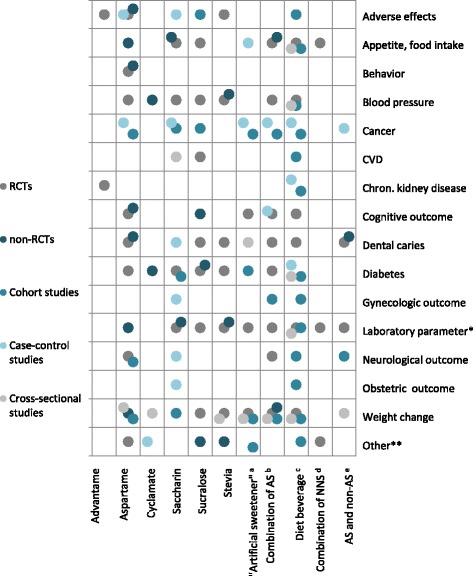
Health outcomes by intervention investigated in primary studies. ^a^ studies, where authors investigated effects of “artificial sweeteners” (no further details for the intervention/exposure is provided); ^b^ authors investigated the combined effects of two or more artificial sweeteners (type of sweeteners is described); ^c^ any type of “diet beverage”, where the type of sweetener is not defined; ^d^ combined effect of AS and NNCS was investigated or the intervention/exposure was described as “non-nutritive sweeteners” (without further details); ^e^ the investigated intervention/exposure is a combination of NNS and other non-sugar sweeteners (e.g. sugar alcohols). * haematological parameters, blood chemistries and hormone levels; **any other health outcome, which couldn’t be classified to any of the above listed categories (e.g. male fertility [289], offspring forearm fractures [290], emotional state [291], analgesia [292] or mortality [293]). Abbreviations: AS, artificial sweeteners; CVD, cardiovascular disease; NNS, non-nutritive sweeteners

Health outcomes by intervention as investigated in primary studies are shown in Fig. 2. We first report short-term outcomes (appetite and short-term food intake), then long-term health outcomes in healthy populations (in alphabetical order: cancer, chronic kidney disease, dental caries, diabetes, headaches, neurocognitive outcomes, obstetric outcomes, weight gain and obesity). Finally, health outcomes in non-healthy populations are described.

### Short-term outcomes

#### Appetite and short term food intake

Eating behavior and metabolic effects due to the exposure to NNSs were investigated in five systematic reviews among other outcomes [20–24]. One review reported evidence for an appetite lowering effect of aspartame, whereas the other reviews reported conflicting evidence for the effects of Stevia and ASs in general on eating behavior.

The primary studies on short-term food intake focused on whether exposure to NNSs enhances the desire for sweet foods and drinks, leading to an increased food intake. From the included 60 primary studies, 32 were small, cross-over RCTs [25–56] with a similar design: the subjects first consumed a “preload”, a food or drink sweetened with either NNSs or with sugar (a nutritive sweetener) or a food or drink which did not contain any sweetener (e.g. water). After a time delay subjects were offered an ad libitum meal and total energy intake was measured.

No effects of NNSs on short-term food intake or subjective awareness of hunger were described in 39 studies (9 parallel RCTs [53, 57–64], 22 cross-over RCTs [25–29, 31, 33–39, 41, 43, 46, 50, 51, 53–56], 7 non-RCTs [45, 65–70] and 1 case-control study [71]); 10 studies described an increased [32, 40, 45, 47, 49, 52, 72–75], while 11 studies described a decreased food intake or appetite [30, 42, 48, 76–83] in the NNSs intervention group as compared to the sugar-receiving or placebo group.

### Long-term health outcomes in healthy populations

#### Cancer

Berry et al. [84] systematically summarized studies on the carcinogenic potential of sucralose and concluded that sucralose does not demonstrate carcinogenic activity even when exposure levels are several orders of magnitude greater than the range of anticipated daily ingestion levels. Another, broadly focused systematic review published in 2015 [85] assessed cancer risk among several other health outcomes. Authors of this review also searched for diet beverage studies, but only narratively summarized their results and concluded that, based on the available data, it was not possible to establish a link between cancer risk and the consumption of ASs.

**Fig. 3:**
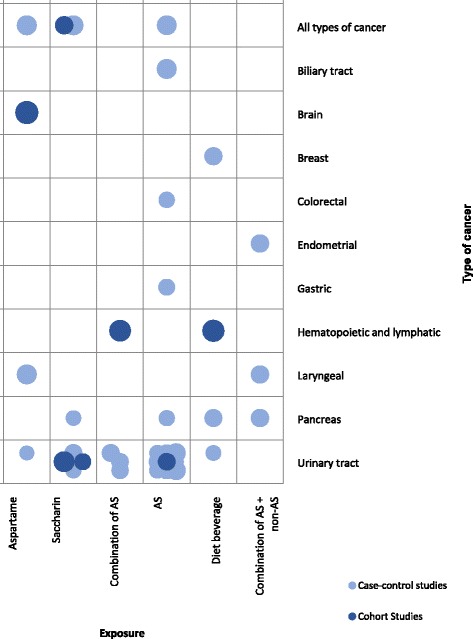
Cancer outcomes by exposure investigated in primary studies

In total, we identified 51 primary studies assessing the association of NNS consumption and cancer risk. The investigated exposure was use of any type of ASs or use of a subtype of ASs (saccharin or aspartame) in 47 studies, while 4 studies investigated exposure to NNCSs. Cancer outcomes by type of exposure as investigated in primary studies are shown in Fig. 3.

Out of the identified 41 case-control studies reporting on the effect of NNSs on cancer, 32 assessed the relationship between NNS consumption and the risk of developing bladder cancer or urinary tract cancer. The results of these studies are controversial: 11 case-control studies describe a positive association between AS/NNS intake and bladder or urinary tract cancer risk [86–96], while 20 report no association [97–116].

Two case-control studies assessed the risk of brain cancer (no association with AS use [117, 118]), 1 study assessed the risk for colorectal cancer (significantly increased with AS use [119]), 2 studies investigated the risk of pancreatic cancer (no association with NNSs [120, 121]), 1 study investigated the risk of breast cancer (no association with AS use [122]) and 4 studies investigated the risk of any type or more types of cancer (no association with NNS use [123–126]).

Three prospective cohort studies investigated the risk of lymphomas or other hematological malignancies [127, 128], 1 assessed the risk of biliary tract cancer [129], 1 assessed cancer incidence in general [130], 1 assessed the risk of tumor multiplicity in treated bladder cancer patients [131], 1 investigated the 5-year survival rate in urinary bladder cancer patients [132], while 2 retrospective cohort studies assessed the risk of bladder cancer [112, 133] (no significant associations were described in either of them).

The cross-sectional study described that breast cancer survivors compared to age-matched controls had significantly lower intakes of NNSs [134].

#### Chronic kidney disease

In a systematic review by Cheungpasitporn et al. [135], the 4 included studies assessed the association between consumption of artificially sweetened soda and chronic kidney disease. The authors concluded that consuming artificially sweetened soda did not increase the risk of chronic kidney disease in high-risk patients.

The primary studies we found on the association of NNS consumption and the risk of developing chronic kidney disease were 3 prospective cohort studies (describing no association [136–138]), 1 case-control study (describing a significant positive association [139]) and 2 cross-sectional studies (one of them indicating a positive association [136, 140]).

#### Dental health (caries)

We found 16 intervention studies (14 RCTs [141–154] and 2 non-RCTs [155, 156]) on the association of an NNS intervention and dental health. Details of these studies are summarized in Table 3.

**Table 3:** Characteristics of studies investigating the effects of non-nutritive sweeteners on dental outcomes

First author, publication year	Study sample (n)	Intervention/Exposure	Control	Outcome	Effect
Interventional studies: randomized controlled trials with parallel-group design					
Beiswanger, 1998 [141]	children (1818)	sugar-free chewing gum containing AS and non-AS	no intervention	development of caries/caries prevalence	decreased development of caries
Lopez de Bocanera, 1999 [142]	both adults and children (32)	a solution/drink with AS	sugared solution/drink	salivary or plaque pH	no effect on pH
Interventional studies: randomized controlled trials with cross-over design					
Brambilla, 2014 [143]	adults (20)	a solution/drink with stevioside	sugared solution/drink	salivary or plaque pH	less acidogenic (increased) pH
Jawale, 2012 [144]	adults (20)	diet soft drink	sugared solution/drink	salivary or plaque pH	less acidogenic (increased) pH
Manning, 1993 [145]	adults (10)	sugar-free chewing gum containing AS and non-AS	sugared chewing gum	salivary or plaque pH	less acidogenic (increased) pH
Mendes de Santa, 2014 [146]	adults (9)	a solution/drink with a combination of NNS	sugared solution/drink	salivary or plaque pH	less acidogenic (increased) pH
Mentes, 2001 [147]	adults (29)	a solution/drink with AS and non-AS	sugared solution/drink	salivary or plaque pH	less acidogenic (increased) pH
Meyerowitz, 1996 [148]	age group not described (14)	a solution/drink with sucralose	sugared solution/drink	salivary or plaque pH	less acidogenic (increased) pH
Park, 1993 [149]	age group not described (5)	sugar-free chewing gum containing sucralose/ ace K	another NNS	salivary or plaque pH	no difference in pH
Park, 1995 [150]	adults (8)	sugar-free chewing gum containing AS or non-AS	sugared chewing gum; no intervention	salivary or plaque pH	less acidogenic (increased) pH
Roos, 2002 [151]	children (17)	diet soft drink	sugared solution/drink	salivary or plaque pH	less acidogenic (increased) pH
Steinberg, 1995 [152]	age group not described (10)	a solution/drink with sucralose	sugared solution/drink	salivary or plaque pH	less acidogenic (increased) pH
Steinberg, 1996 [153]	age group not described (12)	a solution/drink with sucralose	sugared solution/drink	salivary or plaque pH	less acidogenic (increased) pH
Zanela, 2002 [154]	children (T: 200)	a solution/drink with stevioside	chlorhexidine gluconate	amount of plaque formed	less effective in decreasing the amount of plaque formed
Interventional studies: non-randomized controlled trials					
Mühlemann, 1985 [155]	adults (T:2)	a solution/drink with aspartame	sugared solution/drink	salivary or plaque pH	no effect on pH
Syrrakou, 1993 [156]	age group not described (15)	a solution/drink with sucralose	sugared solution/drink	salivary or plaque pH	less acidogenic (increased) pH
Observational studies: case-control studies					
Grenby, 1975 [287]	adults (24)	saccharin instead of sucrose	sugared solution/drink	amount of plaque formed	decreased amount of plaque formed
Observational studies: cross-sectional studies					
Serra-Majem, 1993 [288]	age group not described (893)	AS in regular diet	–	development of caries/caries prevalence	decreased development of caries

*Abbreviations*: *AS* artificial sweetener, *ace K* acesulfame potassium, *n* total number of participants, *non-AS* a non-sugar sweetener other than NNS (e.g. sugar alcohols)

Only two of the studies mentioned above described no differences between intervention and control groups [142, 155]; all other studies described a less acidogenic (increased) oral pH after the intervention as compared to the sugar-containing control.

#### Diabetes

In a systematic review published in 2014 [157], three included publications on 4 cohorts investigated the association between intake of artificially sweetened soft drinks and risk of type-2 diabetes [158–160] using additional information provided by the authors of two of the publications [158, 159]. The review reported an increased risk of diabetes when consuming 330 ml/day of artificially sweetened soft drinks; however, substantial heterogeneity was described among the cohort studies. Also, another systematic review published in 2016 [161] described a positive association between the consumption of artificially sweetened beverages and type-2 diabetes incidence; however, the authors of this review rated their findings as biased.

We found 6 prospective cohort studies (4 with an AS exposure and 2 with a “diet beverage” exposure), 1 retrospective cohort study (with AS exposure) and 1 case-control study (with AS exposure) on the risk of developing diabetes. These studies are summarized in Table 4.

**Table 4:** Cohort studies on the association of AS consumption and risk of developing diabetes

First author, publication year	Study sample	Number of participants	Exposure	Main outcome	Direction of effect
Prospective cohort studies					
Bhuphatiraju, 2013 [165]	female nurses (age 30–55 y) + male health professionals (age 40–75 y)	74,749 + 39,059	ASB	risk of type 2 diabetes	–
deKonig, 2011 [160]	middle-aged (40–75 y) male health care providers	40,389	ASB	incidence of type 2 diabetes	–
Fagherazzi, 2013 [162]	women	66,118	ASB	risk of type 2 diabetes	↑↑
Fagherazzi, 2017 [163]	women	61,440	AS in packets or tablets	risk of type 2 diabetes	↑↑
Palmer, 2008 [285]	women (age 21–69 y)	43,960	diet soft drink	risk of type 2 diabetes	–
Schulze, 2004 [217]	healthy women	91,249	diet soft drink	risk of diabetes	↑
Sakurai, 2014 [286]	men	2037	diet soda	risk of type 2 diabetes	↑↑
Retrospective cohort studies					
Armstrong, 1975 [166]	bladder cancer patients + patients with other cancers	18,733 + 19,709	saccharin	prevalence of diabetes	–
Case-control study					
The Inter Act Consortium, 2013 [164]	type 2 diabetes cases + controls	11,684 + 15,374	artificially sweetened soft drink	incidence of type 2 diabetes	↑

*Abbreviations*: *ASB* artificially sweetened beverage consumption, *y* years, *AS* artificial sweeteners; ↑ means that a positive association was suggested in the study, but this was not significant; ↑↑ means a significant positive association; − means that there was no (significant) difference in the outcome between the intervention and control group

Among the studies investigating the exposure to AS, 2 prospective cohort studies [162, 163] and one case-control study [164] described an increased risk of type-2 diabetes, while 2 prospective [160, 165] and 1 retrospective cohort studies [166] found no association between AS consumption and risk of diabetes. There were no studies investigating diabetes risk in association with NNCS consumption.

#### Headaches

We found 3 RCTs [167–169] with a cross-over design and 2 cohort studies [170, 171] investigating the effect of AS on headaches. These included either healthy populations or populations with a subjectively reported sensitivity to AS or people with a history of migraines. Two of them (one RCT [168] and one cohort study [170]) described a significant positive association, in the others no significant association was found between AS consumption and headaches.

#### Cognitive effects, mental health

RCTs assessing the behavior and mood of essentially healthy children after they were given a preload of either an artificially sweetened or sugar-sweetened food or beverage found no consistent effect of ASs on behavior. Most of the interventional and observational studies investigating the effect of an AS preload on cognitive abilities in healthy children and adults demonstrated that there was no association between cognitive performance, measured by an array of tests, and the intake of ASs in different forms.

Three studies (2 RCTs and 1 cohort study) investigated the effect of AS on depression and described an increased risk of developing depression symptoms or increased severity of symptoms in mood disorder patients [172–174]. In 1 case-control study, consumption of saccharin was significantly positively associated with the risk of Alzheimer’s disease [175].

#### Obstetric outcomes

Three cohort studies investigated the effect of AS consumption and preterm delivery [176–178], two of them describing a significant positive, while one described no association. One case-control study described no association between saccharin use before conception or during pregnancy and spontaneous abortion [179].

#### Weight change

We found 4 systematic reviews addressing the question whether NNS consumption has an unfavorable or favorable effect on body weight [22, 180–182]. Details of these reviews are described in Table 2. Miller et al. [181] indicated, based on data from RCTs, that substituting low-calorie sweeteners (LCS, including NNSs and sugar-alcohols) for calorically dense alternatives resulted in a modest reduction of body weight, body mass index (BMI), fat mass, and waist circumference. Rogers et al. [182] concluded, based on results of relevant RCTs, that low-energy sweetener consumption does not increase body weight. The meta-analysis of observational studies showed a significant positive association between LCS intake and slightly increased BMI, but no association with body weight or fat mass. Pereira et al. [180] concluded that results of the epidemiologic studies are highly inconsistent.

A systematic review [22] focusing on metabolic health effects of AS consumption in pediatric populations identified 3 large cohort studies with long-term follow-up, supporting the existence of an association between ASB (artificially sweetened beverage) consumption and weight gain in children, while 2 other prospective cohort studies described no or an inverse association with obesity. The identified 3 RCTs on children described no differences in weight or BMI between the NNS and the control groups.

Another systematic review [183] focusing on long-term metabolic effects of early NNS consumption concluded that the current evidence of the long-term metabolic effects of NNS exposure during gestation, infancy, and childhood is limited and inconsistent.

We found 31 interventional studies (27 RCTs [58, 61, 62, 74, 77, 184–205] and 4 non-RCTs [67, 68, 79, 206]) and 36 observational studies [158, 159, 202, 203, 207–241] on the effect of NNS consumption on BMI or weight change, including recently published studies, which were not included in the systematic reviews presented above.

Of the 27 RCTs, 14 reported a weight reduction after the intervention with NNSs or diet beverages, 2 reported an increase in weight, while in 11 RCTs no weight change was observed. After subdividing the RCTs according to the type of exposure, we found 15 RCTs with an AS intervention, 8 describing a decrease in body weight after the AS intervention as compared to the (sugar-containing or unmodified) control intervention, 1 describing an increase, while in 6 AS intervention studies no differences were observed between the two groups. There were 3 RCTs with a NNCS (stevia) intervention [187, 204, 242]. None of them described a difference in change of body weight between the intervention and control groups.

Of the 17 prospective cohort studies, 10 described a positive association (either statistically significant or a non-significant trend) between NNS or diet beverage consumption and weight gain/increased BMI [159, 207–211, 216, 218, 237, 239], 3 observed an inverse association [214, 215, 217], while in 4 prospective cohort studies no association [212, 213, 219, 220] between body weight and NNS consumption was found. When investigating the subgroup of prospective cohort studies with a clear AS intervention (8 studies), we found 7 studies describing a positive [208–210, 216, 218, 237, 239] and 1 study describing no association [219] between AS consumption and weight gain/increased BMI. There were no cohort studies with a NNCS intervention reporting on weight gain or obesity.

Of the 17 cross-sectional studies, 12 described a positive [158, 222–226, 229–233, 241], 2 a negative [227, 235] and 3 no association [221, 228, 234] between NNS or diet beverage consumption and weight gain/increased BMI.

### Health outcomes in non-healthy populations (diabetes and hypertension)

There are two main disease groups with a relatively wide literature of NNS intervention studies. In type-1 and type-2 diabetes patients, the effects of NNS use on diabetic control, including, but not limited to, blood glucose levels, postprandial blood glucose, and glycated hemoglobin (HbA1c), are widely investigated. We found 21 interventional studies (13 RCTs [33, 198, 243–253] and 4 non-RCTs [254–257] with an AS intervention, and 4 RCTs [193, 258–260]) and 2 non-RCT with an NNCS intervention [261] on this topic.Most of the studies described no difference in diabetic patients on diabetic control between the NNS intervention and the control group. Some studies investigated the glycemic effects of NNSs in people with insulin resistance and impaired glucose tolerance [204, 206, 262].

The other disease group consists of hypertensive patients, where the role of NNSs in blood pressure control has been investigated. We found 9 RCTs [187, 193, 242, 259, 263–267], 4 prospective cohort studies [268–271], 1 case-control study [272] and 1 cross-sectional study [273] on this question, with controversial results.

## Discussion

### Summary of findings

Overall the evidence for health outcomes of AS is inconsistent and there are numerous gaps in the evidence base. In healthy subjects, appetite and short term food intake, risk of cancer, risk of diabetes, risk of dental caries, weight gain and risk of obesity were the most investigated health outcomes.

In case of the health outcome appetite and short term food intake, a majority of studies were short interventions with a cross-over design. A smaller part were randomized controlled trials with an intervention duration of 4 weeks up to 18 months. In case of the longer interventions, the type and dosage of the NNS was often not defined.

Bladder cancer and cancer of the urinary tract were investigated in multiple studies. For this type of cancer a systematic review may provide conclusive evidence. Most of the studies on urinary tract cancer investigated effects of artificial sweeteners in general; a smaller number investigated the effects of aspartame or saccharin. Other types of cancer were investigated in only one or a low number of studies.

We also found several studies on the role of NNS in dental caries prevention. Included studies suggested that stevia in chewing gum or NNCS beverages instead of sugar-sweetened beverages may be an effective tool for dental caries prevention. However, it has to be mentioned, that while sugar alcohols are widely used in chewing gums for caries prevention, and the literature on their effects is broad, the effects of NNCS on dental caries is investigated in a limited number of studies. In addition, in these studies, NNCSs were often combined with sugar alcohols. It would be interesting to see more comparative studies on the effectiveness of NNCS alone versus other interventions in influencing dental plaque pH; or studies with a longer intervention period and follow-up.

The effect of NNS on risk of diabetes was investigated in a limited number of cohort studies. These studies mainly focused on artificially sweetened beverage or diet beverage consumption and described different directions of effect. Further studies, focusing on special types of NNS (also including NNCS), are required.

Intervention studies on weight change focused mainly on the question whether NNS can be efficiently used in weight management. As part of weight loss intervention programs, more intervention studies would be required, to investigate the effects of NNS alone on body weight in both overweight, obese and normal-weight subjects. This would be especially important, since it is very difficult in observational studies to evaluate causality between NNS consumption and BMI/weight change and therefore results of these studies have to be interpreted with caution. A positive association between NNS consumption and weight gain in observational studies may be the consequence of and not the reason for overweight and obesity. Moreover, other factors, such as population characteristics, may influence the results of observational studies.

In subjects with diabetes, the effects of NNS were investigated mainly on glycemic control. Because of the heterogeneous, if not contradictory results, a thorough analysis of these findings in a full-fledged systematic review including meta-analyses, subgroup and sensitivity analyses is needed and might help to resolve some of the ongoing uncertainties. Further studies on long-term patient-relevant outcomes in diabetes are required.

The effect of NNS on lowering blood pressure in hypertensive patients should also be analyzed in a high quality systematic review and meta-analysis.

Regarding NNCS, although Stevia is increasingly used as a sweetener, the number of studies on its health effects is limited as of now. Studies investigating the effects of NNCS on cancer or diabetes risk are completely lacking, while there are only few studies on weight gain and obesity risk. Clearly, there is a need for further research.

Eligible NNS not addressed by any of the included primary studies were: neotame, alitame, neohesperidin DC, thaumatin and brazzein.

### Strength and limitations of this scoping review

The strength of our scoping review is its inclusion of all types of primary studies and systematic reviews which investigate any health effect of any NNS in any population. We are therefore able to present a comprehensive overview of the available scientific evidence on health effects of NNS.

Our scoping review might be limited by the following factors. Firstly, the literature search was conducted in three major and comprehensive databases, but we might have inadvertently missed relevant studies listed in other databases. Secondly, the title abstract screening was conducted by one reviewer who might have inadvertently excluded relevant studies at the first stage of the screening. This limitation might be evened out by conducting the literature search in two steps. In the second step, relevant references for both topics were identified and the chances for including all relevant references in our review were increased.

Detailed assessment of the study quality is not covered in a scoping review and was not conducted in the context of our scoping review. Therefore, information gathered on the health outcome includes only its direction of effect but no information on the internal or external validity of the study results.

### Discussion of findings in light of other evidence summaries

In our scoping review we found a large number of studies of different designs, investigating effects of different types of NNS in different populations on a variety of health outcomes.

Systematic reviews to summarize the available evidence are already available (Table 2). However, they often have methodological limitations (e.g. language limitation of the search, electronic search in only one database, etc.) or a narrow scope.

There are systematic reviews, which also included key words for “diet soda” and “diet beverage” in their search strategy. There are several, primarily observational studies, where the exposure is defined as “diet”, which may indicate NNS-containing beverages, but further details are often not provided. Therefore, it is clearly a challenge when trying to synthesize the evidence to decide, how to deal with studies describing the intervention/exposure as “diet beverage”, “diet drink” or “diet soda” only. We also included such studies in this scoping review; however, it has to be mentioned that we did not include specific search terms for “diet” beverages/sodas in our search strategy, therefore the list of studies reporting on the effects of diet beverage etc. may be incomplete.

### Implications of findings for practice, policy and future research

Current evidence demonstrates that there is a need for both further primary research and high quality comprehensive systematic reviews including meta-analyses, to inform future recommendations about the health benefits and risks of NNS to advise and support health care practice and public health decision-making.

This scoping review highlights the need for studies which investigate the long-term effects of individual sweeteners on some of the less well-researched health outcomes (e.g. headaches, depression or other mood disorders, Alzheimer’s disease, risk of preterm delivery). Future studies need to be rigorous in design and conduct, with well-defined interventions (providing information on type and dosage of the non-nutritive sweetener) and controls. Study reports should include detailed descriptions of all methodological aspects to enable proper interpretation of the results.

Systematic reviews are required for health outcomes with a large number of primary studies, but without conclusive evidence (e.g. appetite and short term food intake, risk of cancer, dental caries, risk of diabetes, glycaemic control in subjects with diabetes and blood pressure control in hypertensive patients) to support the formulation of recommendations and to be able to decide whether further, well-designed primary studies are required.

## Conclusions

There are numerous gaps in evidence related to the health effects of NNS in both healthy and non-healthy populations. In healthy subjects appetite and short term food intake, risk of cancer, risk of diabetes, risk of dental caries are the most investigated health outcomes, all of them without any conclusive evidence. There is a need for well-conducted systematic reviews to quantitatively summarize results and assess their validity. Besides, there are numerous health outcomes, like incidence of headaches in association with NNS consumption, depression, Alzheimer’s disease, risk of preterm delivery, behavioural effects, cardiovascular effects or risk of chronic kidney disease, which were investigated in only few studies and further research activity is needed. A systematic review may also help to enable formulating recommendations for subjects with diabetes and hypertension on using NNS.

## Abbreviations

AS: Artificial sweeteners
EFSA: European Food Safety Authority
FDA: Food and Drug Administration
LCS: Low-calorie sweeteners
MeSH: Medical Subject Headings
NNCS: Natural, non-caloric sweetener
NNS: Of non-nutritive sweetener
RCT: Randomized controlled trial
